# Temporal decline in diarrhea episodes and mortality in Kiribati children two years following rotavirus vaccine introduction, despite high malnutrition rates: a retrospective review

**DOI:** 10.1186/s12879-020-4874-6

**Published:** 2020-03-12

**Authors:** Jana Lai, Cattram Nguyen, Beia Tabwaia, Agnes Nikuata, Nikarawa Baueri, Eretii Timeon, Mohammed Diaaldeen, Tinai Iuta, Murat Hakan Ozturk, Aaron Moore, Alice Hall, Batmunkh Nyambat, Stephanie Davis, Ataur Rahman, Wendy Erasmus, Kimberley Fox, Fiona Russell

**Affiliations:** 1 Murdoch Children’s Research Institute, Melbourne, Australia; 2 Australian National University, Canberra, Australia; 3 Department of Paediatrics, The University of Melbourne, Melbourne, Australia; 4 Ministry of Health and Medical Services, Tarawa, Kiribati; 5 World Health Organization, Tarawa, Kiribati; 6 UNICEF, Tarawa, Kiribati; 7 UNICEF Pacific, Suva, Fiji; 8 UNICEF Australia, Sydney, Australia; 9 WHO Regional Office for the Western Pacific, Manila, Philippines; 10 Center for Disease Control and Prevention, Atlanta, USA; 11 Centre for International Child Health, Dept. of Paediatrics, The University of Melbourne, Melbourne, Australia

**Keywords:** Rotavirus vaccine, Diarrheal disease, Severe acute malnutrition, Kiribati, Hospital

## Abstract

**Background:**

Kiribati introduced rotavirus vaccine in 2015. To estimate the impact of rotavirus vaccine on acute gastroenteritis (AGE) and severe acute malnutrition (SAM) among children under 5 in Kiribati, a retrospective review of inpatient and outpatient AGE and hospitalized SAM was undertaken.

**Methods:**

Inpatient data for admissions and hospital deaths due to AGE, SAM and all-causes were collected for children under 5 from all hospitals on the main island, Tarawa, from January 2010–December 2013 (pre-rotavirus vaccine) and January 2016–September 2017 (post-rotavirus vaccine). National outpatient diarrhea data were collected from January 2010 to August 2017 for under 5. An interrupted time-series analysis was undertaken to estimate the effect of rotavirus vaccine on the rates of inpatient and outpatient AGE, inpatient SAM; and inpatient case fatality rates for AGE and SAM, were calculated pre- and post-rotavirus vaccine introduction.

**Results:**

The incidence rate of AGE admissions from Tarawa and national AGE outpatient presentations significantly declined by 37 and 44%, respectively, 2 years following rotavirus vaccine introduction. There was a significant decline in the percentage of AGE contributing to all-cause under 5 admissions (12·8% vs. 7·2%, *p* < 0·001) and all-cause under-five mortality (15·9% vs. 5·7%, *p* = 0·006) pre- and post-rotavirus vaccine introduction. The estimated incidence rate of inpatient SAM decreased by 24% in under 5 s, 2 years following rotavirus vaccine introduction.

**Conclusions:**

AGE morbidity and mortality and hospitalized SAM rates have declined following rotavirus vaccine introduction in Kiribati children.

## Background

Rotavirus is the most common cause of severe diarrheal disease in children worldwide [1] and kills approximately 215,000 children each year [1]. Almost all unvaccinated children will have experienced one or more rotavirus diarrheal episodes, regardless of their living conditions, by the age of five [2]. About one third to half of hospitalized cases of diarrheal illness in under 5 s are associated with rotavirus, with a peak incidence in children 6–24 months old [3, 4]. In temperate climates, rotavirus diarrhea occurs in seasonal peaks during cooler months, whilst in island populations rotavirus diarrhea is constant throughout the year with a moderate peak in the cooler dry months [3, 5].

In 2009, the World Health Organization (WHO) recommended all infants be routinely immunized with rotavirus vaccine to prevent rotavirus disease [6]. Currently licenced rotavirus vaccines (Rotarix®, GSK, RotaTeq®, Merck, ROTASIIL®, Serum Institute of India, ROTAVAC®, Bharat Biotech) are safe and effective against rotavirus acute gastroenteritis (AGE) [7–11]. From a global review, the median percentage reduction in AGE hospitalizations overall was 38% and for those rotavirus AGE hospitalizations and emergency department visits an overall reduction of 67% were observed [12], with greater vaccine coverage leading to greater reductions [13]. The effectiveness of rotavirus vaccine in gastroenteritis-related hospitalizations appears to be lower in low-income countries compared with high-income countries (~ 50% vs. 98%) [14]. A reason for this discrepancy may be due to poorer immunological responses to this oral vaccine due to the effect of malnutrition on gut function [15] or the inhibitory effect of maternal antibodies and co-administration of other vaccines such as oral poliovirus vaccine [16]. A study from Malawi found that stunted infants less than 12 months old had a non-significant lower vaccine effectiveness compared with their well-nourished counterparts [14]. Therefore understanding the situation of malnutrition is important when assessing the impact of rotavirus vaccine in a population.

Few low- or middle-income countries in the Asia-Pacific region have introduced rotavirus vaccine into their national immunization schedules, despite rotavirus causing 43.3% of childhood diarrhea admissions from 2008 to 2016 [15]. Kiribati has one of the highest child mortality rates in the Pacific and, due to substantial challenges with provision of safe drinking water and effective sanitation [17]. Kiribati is prone to regular AGE outbreaks and has high rates of childhood malnutrition [17]. In Kiribati, AGE is estimated to cause 10.7% of all deaths in under 5 s [18]. In 2002, 11% of all hospitalizations in children under four years were due to AGE [17]. Acute respiratory disease and diarrhea have been shown to be common causes of admission in children under 15 years in Kiribati, with malnutrition as an important contributor to poor health [17]. In 2002, malnutrition contributed to 0.8 and 0.7% of all reported cases of disease affecting children under 1 year and 1–4 years, respectively [17]. Prior to rotavirus vaccine introduction, Kiribati experienced large diarrheal outbreaks in 2013 and 2014, with up to 70% of cases affecting children under 5. The 2013 outbreak occurred in July, with 81% of stool specimens testing positive for rotavirus. During that outbreak, 1118 of the reported cases occurred in South Tarawa with 108 hospitalizations and six deaths of children under 5 [19]. The 2014 outbreak occurred between September and October 2014, affecting 2513 children, inclusive of seven deaths. Sixty-six percent of stool samples tested in the 2014 outbreak were positive for rotavirus. To address this high burden of AGE, Kiribati introduced a comprehensive package to improve child survival in August 2015, which included adding rotavirus vaccine to their national immunization schedule. The aim of this study was to estimate the effect of rotavirus vaccine on AGE and severe acute malnutrition (SAM) rates in young children in Kiribati.

## Methods

### Study location

Kiribati is comprised of 33 atolls and low-lying reefs, widely scattered along the Equator in the central Pacific Ocean with a population of approximately 110,000 (according to the country’s 2015 census), with about 11,000 children aged under 5 [17]. In the Pacific region, Kiribati has the lowest level of development with regards to mortality, morbidity, living conditions and per capita Gross Domestic Product, contributing to its least developed country status [17]. Health care is provided free of charge. The island of Tarawa is home to approximately 60% of the total population of Kiribati. Tarawa has a total area of 500 km^2^, with a well-established road running the length of the island, making health care readily accessible to the island population. Patients who require further care are either referred to or seek care at one of the two hospitals on Tarawa: Betio Hospital in Betio or Tungaru Central Hospital (TCH), in Bikenibeu. TCH is the main hospital for Tarawa and also the referral hospital for both Betio Hospital and the outer islands. Rotavirus vaccine (Rotarix©, GSK) was introduced in August 2015 as a two-dose schedule at six and 14 weeks of age. The second dose coverage for rotavirus vaccine was 79% in 2016 and 91% in 2017 [20].

### Study population

Hospitalized AGE cases were defined as any child aged under 5, with a primary discharge diagnosis of acute diarrhea or gastroenteritis admitted to TCH or Betio Hospital from January 2010 to September 2017. Cases of chronic or persistent diarrhea, bloody diarrhea, and dysentery, recorded at discharge, were excluded. Outpatient diarrhea cases were all acute diarrhea cases reported by clinicians from hospitals and clinics throughout the country to the Health Information Unit (HIU) of Ministry of Health and Medical Services (MHMS).

Hospitalized SAM cases were defined as any child under 5, with a primary discharge diagnosis of severe malnutrition, malnutrition, failure to thrive, very low weight, marasmus, or kwashiorkor admitted to TCH or Betio Hospital from January 2010 to September 2017.

### Data collection

Inpatient AGE and SAM data were collected for all under 5 hospitalizations at TCH. Primary discharge diagnosis data were collected from electronic records for the time periods January 2010–December 2013 and January 2016–September 2017. Variables collected were date of birth, date of admission, date of discharge, sex, residence, discharge diagnosis, and outcome (dead/alive).

For outpatient AGE cases in under 5 s, electronic data of total monthly counts of diarrhea/AGE were supplied from the HIU of MHMS and were collected for the time period January 2010–September 2017. These data were reported to HIU on a monthly basis from the clinics and outpatient departments throughout Kiribati. Individual case information and outcome were not available for this data set.

Prior to rotavirus vaccine introduction, rotavirus testing was only performed on a limited number of samples to confirm a diarrhea outbreak. Since 2015, rapid testing has been available at TCH and only used for diarrhea outbreak confirmation. Available rotavirus testing data was collected from the clinical laboratory located in TCH.

### Data analysis

Patient characteristics for both AGE and SAM were compared in pre- and post-rotavirus vaccine periods using chi-square tests. The pre-rotavirus vaccine period was defined as January 2010 to December 2013 and the post-rotavirus vaccine period was defined as January 2016 to September 2017. TCH catchment population was defined as children under 5 residing on Tarawa for monthly inpatient incidence calculations. Hence, only hospitalized AGE and SAM cases residing on Tarawa were included in the population-based analysis for hospitalized cases. For outpatient AGE cases, national population data were used as denominators to calculate the incidence of AGE nationwide. The population denominator data were obtained from the official 2010 census. The age groups in the 2010 census relevant for this review were less than 12 months, 12–23 months, and total under 5. We used 75% of the total under 5 population as the number of children aged 24–59 months old. A 2.2% growth rate was applied per annum to the 2010 census data to calculate the 2011–2017 under 5 population.

An interrupted time series analysis was used to estimate vaccine impact on both inpatient and outpatient AGE and inpatient SAM. The intervening years of 2014–2015 were excluded from analysis due to the hospital database transitioning to a new electronic recording system and data being unreliable during this time period, as evidenced by a 20% discrepancy in AGE counts between the paper-based ward registers and the electronic hospitalization system. In addition, the known outpatient AGE outbreaks were not co-incident with an increase in AGE admissions over the same time period, so the completeness of inpatient data captured during 2014–2015 was suboptimal.

Poisson regression models were fitted to monthly inpatient/outpatient counts. The interrupted time series models included a parameter for time (months since review commencement), a binary variable representing the introduction of the vaccine and an interaction between vaccine introduction and time, to allow for a change in the slope of the trend. The model included a scaling adjustment to handle over-dispersion. The models for AGE also adjusted for outbreaks that occurred in July 2013 and September 2014. To enable estimation of rates, the models included the logarithm of the population estimates as an offset variable. To calculate percentage declines, we estimated counterfactual rates in the absence of vaccine. This was done by fitting the model to the pre-vaccine data and then extrapolating the trend out to the post-vaccine period. The percentage reductions were estimated by comparing the extrapolated/counterfactual values to the model predictions fitted to the observed post-rotavirus vaccine data. All analyses were done in Stata 15.1 (StataCorp, US).

## Results

Post-rotavirus vaccine introduction, the median age of AGE admissions significantly increased from 13.8 to 17.7 months of age (Table 1). In addition, the percentage of AGE contributing to all-cause admissions declined by 43.7% (12.8% vs. 7.2%, *p* < 0.001), and by 64.2% for all-cause mortality (15.9% vs. 5.7%, *p* = 0.006), post-rotavirus vaccine introduction.

**Table 1:** Characteristics of AGE^1^ hospitalizations in children aged under-five pre- (January 2010–December 2013) and post-rotavirus vaccine (January 2016–September 2017) introduction, TCH^2^, Kiribati

Characteristics	Pre-vaccine *n* = 431	Post-vaccine *n* = 102	*p*-value
Median age in months, (IQR)^3^	13.8 (9.5–20.1)	17.7 (11.1–24.3)	< 0.001
Sex (male), n (%)	251 (58.2%)	64 (62.7%)	0.41
Case fatality, n (%)	34 (7.9%)^4^	7 (6.9%)	0.74
All-cause admissions due to AGE^1^, n/N (%)	431/3361 (12.8%)	102/1407 (7.2%)	< 0.001
All-cause mortality due to AGE^1^, n/N (%)	34/213 (15.9%)^4^	7/122 (5.7%)	0.006

^1^*AGE* Acute gastroenteritis

^2^*TCH* Tungaru Central Hospital

^3^*IQR* Interquartile range

^4^ Outcome of two cases unknown

**Fig. 1:**
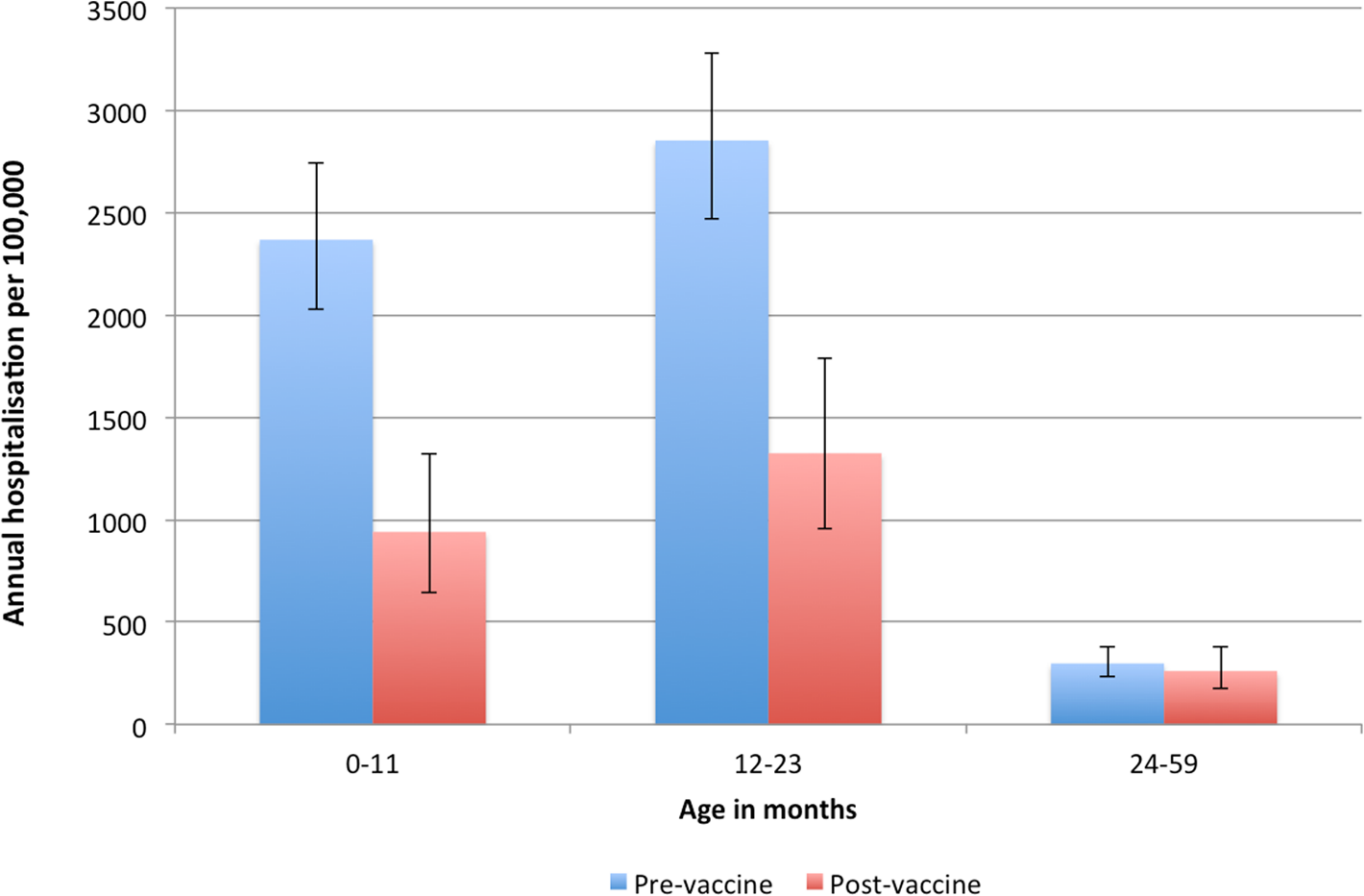
Annual incidence rate of under 5 AGE^1^ admissions to TCH^2^ in the pre- (January 2010–December 2013) and post-rotavirus vaccine (January 2016–September 2017) periods^3^, Tarawa, Kiribati. ^1^AGE = acute gastroenteritis. ^2^ TCH = Tungaru Central Hospital. ^3^ Rotavirus vaccine was introduced in August 2015

The incidence of hospitalized AGE in children both less than 1 year and 1–2 years old in the pre/post-rotavirus vaccine era were significantly different (Fig. 1). However the incidence of hospitalized AGE in children older than 2 years in the pre/post-rotavirus vaccine era were similar (pre-vaccine, 299 per 100,000 (95%CI 232–379) vs. post-vaccine, 261 per 100,000 (95%CI 174–378).

In the early post-rotavirus vaccine introduction period, there was one reported diarrheal outbreak in South Tarawa from mid-August 2016 to the end of September 2016, with few pediatric inpatient and outpatient cases. Eighty-seven samples collected from under 5 s admitted to TCH were tested for rotavirus in the post-vaccine period. Of those, 38 (44%) were positive for rotavirus, with 20 (53%) from children that were age-eligible for vaccination.

**Fig. 2:**
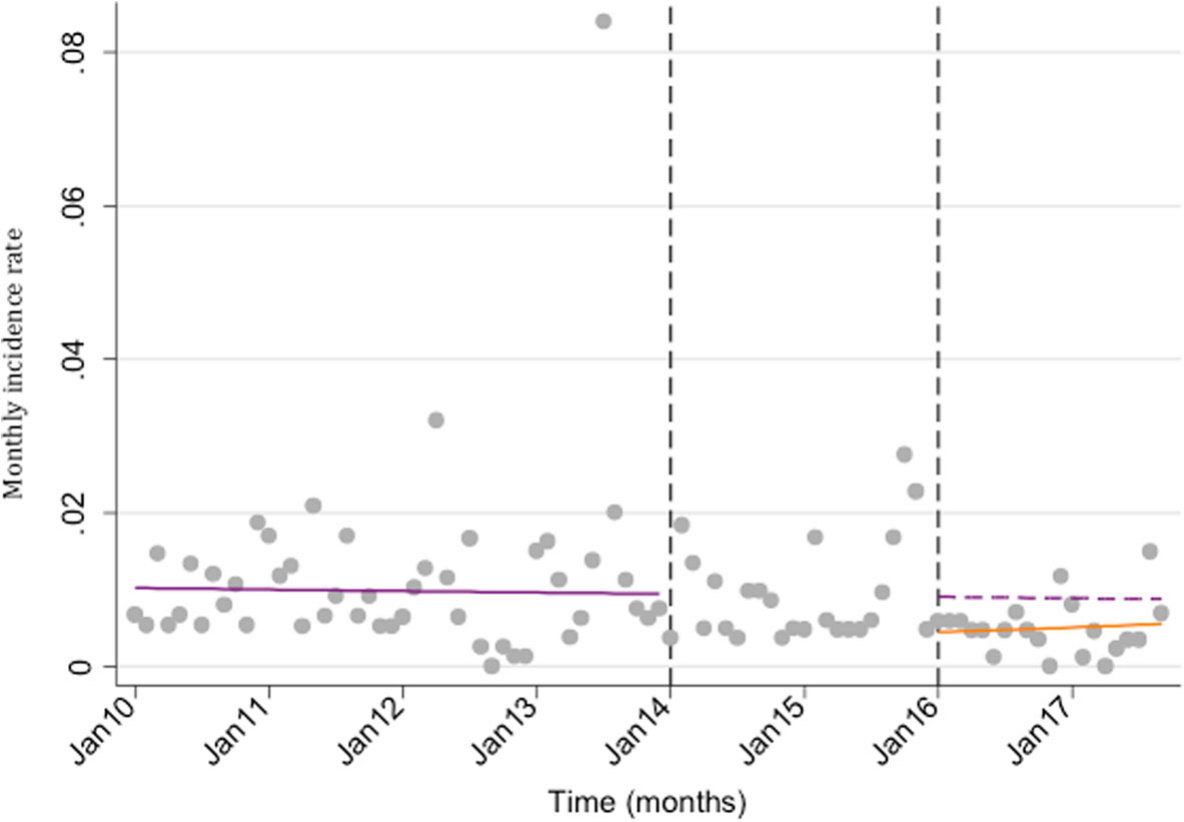
Interrupted time series analysis of the monthly incidence rate of under 5 AGE^1^ admissions in the pre- (January 2010–December 2013) and post-rotavirus vaccine (January 2016–September 2017) periods^2^, Tarawa, Kiribati. ^1^AGE = acute gastroenteritis. ^2^ Rotavirus vaccine was introduced in August 2015^.^ Legend: The purple solid line is the pre-rotavirus vaccine trend based on the model fitted to the observed data in the pre- vaccine period; the purple dashed line is extrapolated from the model fitted to the pre- vaccine data and represents the counterfactual trend that would be expected if the vaccine had not been introduced; the orange solid line is the post-vaccine trend based on the model fitted to the observed data two years post- vaccine introduction

The monthly incidence rate of AGE admissions to TCH in cases under 5 was compared in the pre- and post-rotavirus vaccine periods. There was an estimated 37% decline in AGE admissions in under 5 s, two years after vaccine introduction (Fig. 2).

**Fig. 3:**
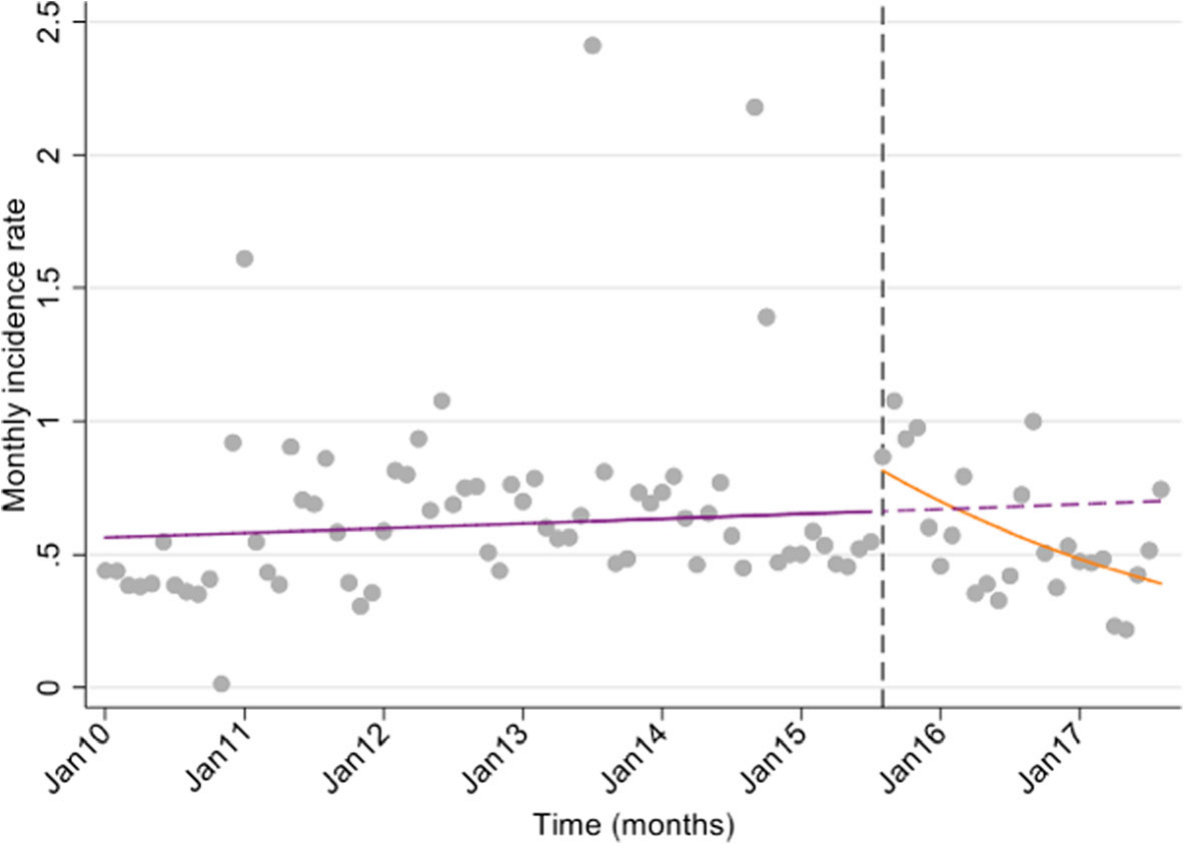
Interrupted time series analysis of the monthly incidence rate of under 5 with outpatient AGE^1^ during the pre- (January 2010–August 2015) and post-rotavirus vaccine (September 2015–September 2017) periods^2^, Kiribati. ^1^AGE = acute gastroenteritis. ^2^ Vertical line on figure denotes the month rotavirus vaccine was introduced. Legend: The purple solid line is the pre-rotavirus vaccine trend based on the model fitted to the observed data in the pre-vaccine period; the purple dashed line is extrapolated from the model fitted to the pre-vaccine data and represents the counterfactual trend that would be expected if the vaccine had not been introduced; the orange solid line is the post-vaccine trend based on the model fitted to the observed data post- vaccine introduction

There was an estimated 44% decline in national outpatient diarrheal presentations in under 5 s in the two-year period post-rotavirus vaccine introduction (Fig. 3).

Post-vaccine introduction, the median age of SAM cases increased significantly compared to the pre-period, as did the contribution of SAM to all-cause admissions (Table 2).

**Table 2:** Characteristics of SAM^1^ hospitalizations in under-fives pre- (January 2010–December 2013) and post-rotavirus vaccine (January 2016–September 2017) introduction, TCH^2^, Kiribati

Characteristics	Pre-vaccine *n* = 163	Post-vaccine *n* = 138	*p*-value
Median age in months, (IQR)^3^	13.0 (8.9–18.1)	16.0 (11.8–23.8)	< 0.001
Sex (male), n (%)	80 (49.1%)	84 (60.8%)	0.07
Case fatality, n (%)	33 (20.2%)^4^	16 (11.6%)	0.08
All-cause admissions due to SAM^1^, n/N (%)	163/3198 (5.1%)	138/1269 (10.9%)	< 0.001
All-cause mortality due to SAM^1^, n/N (%)	33/213 (15.5%)^4^	16/122 (13.1%)	0.55

^1^*SAM* Severe acute malnutrition

^2^*TCH* Tungaru Central Hospital

^3^*IQR* Inter-quartile range

^4^Outcome of one case unknown

**Fig. 4:**
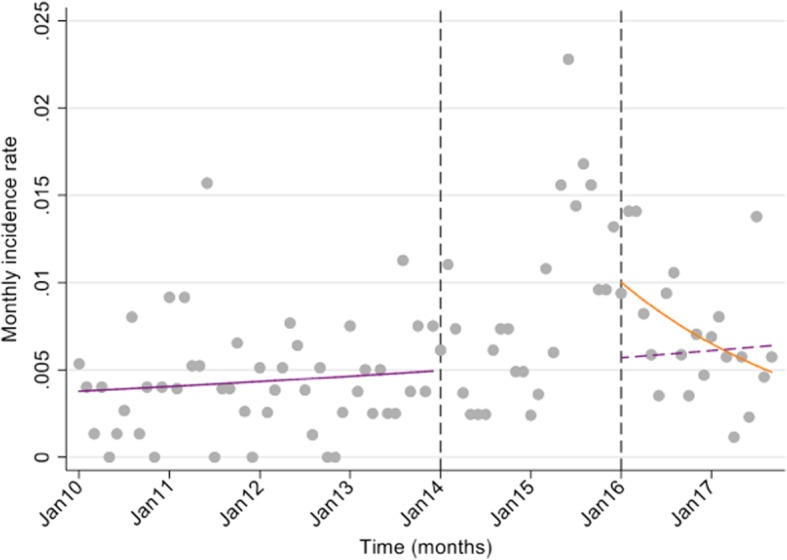
Interrupted time series analysis of the monthly incidence rate of under 5 s admitted with SAM^1^ in the pre- (January 2010–December 2013) and post-rotavirus vaccine (January 2016–September 2017) periods^3^, TCH^2^, Kiribati**.**^1^ SAM = severe acute malnutrition. ^2^ TCH = Tungaru Central Hospital. ^3^ Vertical line on figure denotes the month rotavirus vaccine was introduced. Legend: The purple solid line is the pre-rotavirus vaccine trend based on the model fitted to the observed data in the pre-vaccine period; the purple dashed line is extrapolated from the model fitted to the pre-vaccine data and represents the counterfactual trend that would be expected if the vaccine had not been introduced; the orange solid line is the post-vaccine trend based on the model fitted to the observed data two years post-vaccine introduction

There was a decrease of 24% in the estimated monthly incidence rate of SAM admissions in under 5 s at TCH in the two-year period post-rotavirus vaccine introduction (Fig. 4).

## Discussion

Close to two years post-rotavirus vaccine introduction, we found an estimated 37% decline in AGE admissions for children under 5. Findings are consistent with a recent global review of all-cause AGE for the first 10 years since rotavirus vaccine licensure [21], with median percentage reductions of 38 and 46% in AGE hospitalizations overall and in countries with high child mortality rates respectively [21]. There are few studies from similar settings in the Pacific. An unpublished study from Fiji found a 39% decline in all-cause AGE hospitalisations in under 5 s, five years following the introduction of rotavirus vaccine (pers. comm. F. Russell).

Differences in effect sizes on rotavirus impact on AGE between countries are likely due to differences in health seeking behaviour, access to health care, admission criteria, other causes of AGE, rotavirus vaccine coverage, time since introduction, interactions with other vaccines, effect of maternal antibodies, the presence of comorbidities and environmental factors [15, 16]. As our estimates are based on a single observed time point, close to two years post-rotavirus vaccine introduction, further follow up is recommended to measure trends over a longer period. In addition, we were unable to verify the completeness or accuracy of the data sources. However, we found that this temporal decline in AGE admissions did not occur in children greater than two years old, an age group ineligible for vaccine. We would not expect a decline to occur in this unvaccinated older age group close to two years post-vaccine introduction as herd protection was unlikely to manifest so early following vaccine introduction [20]. Furthermore, the median age of AGE hospitalizations increased in the post-rotavirus vaccine introduction period, suggesting infants who were age-eligible for vaccine were protected (direct effects), but older age-ineligible children were not yet protected by herd immunity. This provides supportive evidence the temporal decline in AGE hospitalizations following rotavirus introduction is due to the direct effects of rotavirus vaccine.

Six years prior to rotavirus vaccine introduction, Kiribati experienced repeated diarrheal outbreaks, with up to 70% of cases affecting children under 5. Following rotavirus vaccine introduction, there was one reported diarrheal outbreak in South Tarawa from mid-August 2016 to the end of September 2016. Comparing the outbreaks in 2013, 2014 and 2016, the 2016 outbreak had fewer inpatient and outpatients cases than either of the two previous outbreaks. Moreover, we found a 44% reduction (12.8 to 7.2%, *p* < 0·001) in the contribution of AGE to all-cause childhood admissions at TCH. However, 52% of rotavirus positive diarrheal samples tested in the post-vaccine period were collected from vaccine age-eligible children. It was unknown whether these rotavirus diarrheal cases were vaccine failures or if the children were unvaccinated, as their vaccination status was not recorded. Nevertheless, we expect that the benefits of rotavirus vaccine are yet to fully manifest, as the population effects are likely to unfold over subsequent years.

Further supportive evidence the temporal decline in AGE is due to rotavirus vaccine was the 44% decline in national AGE outpatient presentations close to two years post-rotavirus vaccine introduction. Possible alternate reasons for this decline may be a reduction in presentations to outpatient clinics due to children being treated at home, decline in reporting rates from clinics, or rotavirus not in circulation during the post-vaccine period. Changes in health-seeking behaviour would be unlikely, as health care is publicly funded and access to care likely improved over this time due to the completion of the main road linking the full length of the island of Tarawa. Clinic reporting rates have improved since rotavirus vaccine introduction with the establishment of report monitoring by the HIU of MHMS (pers. comm K. Corbett, Chief Health Information Officer). The most likely explanation would be the reduction of rotavirus transmission in the community through introduction of rotavirus vaccine, or no importation of rotavirus in the community. Therefore, it is important to continue surveillance of diarrheal disease and undertake further evaluations in the future to ensure diarrheal rates continue to decline.

Prior to rotavirus vaccine introduction, diarrhea in Kiribati was a major contributor to the under 5 mortality rate of 56 per 1000 live births [22]. Following rotavirus vaccine introduction, we found a 64.2% reduction in all-cause childhood mortality due to AGE which is much higher than the median reduction of 40% reported in the global review post-rotavirus vaccine introduction, in high child mortality settings [21]. The additive effect of other interventions in the broad integrated Child Survival Package in Kiribati rather than rotavirus vaccine alone is likely to have contributed to the larger reduction observed. These interventions included high-impact, low-cost solutions linking immunization with reproductive, maternal, newborn, child and adolescent health, the Integrated Management of Child Illness, and nutrition programmes that actively promote exclusive breastfeeding and vitamin A supplementation; working jointly with water and sanitation interventions; and building an uninterrupted supply of oral rehydration salts and zinc for the treatment of AGE. These interventions would confound the impact of rotavirus vaccine, particularly for mortality.

Hospitalized SAM cases decreased by 24% two years following vaccine introduction. However, the percentage of SAM contributing to all-cause admissions significantly increased during the post-vaccine period. SAM is often listed as a comorbidity on the discharge diagnoses with AGE or other conditions, rather than the primary cause of admission. With a decline in diarrhea, it is likely that this increase in SAM’s contribution to all-cause admissions is due to SAM no longer being identified as a comorbidity but rather the primary reason for hospitalization, resulting in an unmasking effect. Furthermore, the improved identification, referral and case management of SAM cases due to the child survival package being introduced at a similar time may have affected the findings. Nonetheless, our results highlight the high burden of SAM in Kiribati. Even though the burden of SAM was still high in the post-rotavirus vaccine evaluation, the incidence, case fatality ratio and contribution of SAM deaths to all-cause hospital mortality in under 5 s declined in the post-rotavirus vaccine period, suggestive of improvements in case management. However, the true burden of SAM is unknown, as most SAM cases are not hospitalized, rotavirus AGE may be occasionally incorrectly coded as SAM and AGE also contributes to transient SAM. Thus the SAM burden we found in our study is likely to be an underestimate of the true burden in Kiribati. In other studies, the presence of SAM has been shown to increase the odds of mortality in children suffering diarrhea as a primary diagnosis [23–26]. However, we could not verify this in our study, as individual medical records were not available for review.

In addition to the limitations we have already outlined, causation between rotavirus vaccine introduction and the decline in outpatient and inpatient AGE cannot be established. Also we did not include a sensitivity analysis on the effects of excluding the 2014–2015 data on the final results or take into account any possible transiency of the population or misreporting of residence that potentially could have affected the results. We were unable to adjust for confounders, such as seasonality and breastfeeding. However, we have outlined compelling evidence to suggest a temporal relationship between vaccine introduction and declines in AGE due to the differential age effect. We were unable to calculate vaccine effectiveness, as rotavirus testing is not routinely performed on AGE samples and individual vaccination status was not known for each case.

## Conclusions

Following the introduction of rotavirus vaccine in Kiribati, there have been declines in morbidity and mortality of AGE and inpatient SAM. These declines are likely due to rotavirus vaccine and the integrated child health package introduced. This reduction in AGE is likely to lead to a substantial impact on health worker workload. Continued monitoring of trends in diarrheal disease in children under 5 and SAM is required to document the full impact of rotavirus vaccination and other child health interventions. These data will be needed to support the continued use of these interventions in Kiribati and support their implementation elsewhere.

## Data Availability

The datasets used and analysed during the current study are not publicly available as they are the property of the Ministry of Health and Medical Services of Kiribati, but are available from the corresponding author on reasonable request.

## Abbreviations

AGE: Acute gastroenteritis
HIU: Health Information Unit
IMCI: Integrated management of childhood illness
MHMS: Ministry of health and medical services
ORS: Oral rehydration solution
RMNCAH: Reproductive, maternal, newborn, child and adolescent health
SAM: Severe acute malnutrition
TCH: Tungaru central hospital
UNICEF: United nations children’s fund
WHO: World health organization

## References

[1] Troeger C, Khalil IA, Rao PC, Cao S, Blacker BF, Ahmed T, Armah G, Bines JE, Brewer TG, Colombara DV, Kang G, Kirkpatrick BD, Kirkwood CD, Mwenda JM, Parashar UD, Pertri WA, Riddle MS, Steele D, Thompson RL, Walson JL, Sanders JW, Mokdad AH, Murray CJL, Hay SI, Reiner RC (2018). Rotavirus vaccination and the global burden of rotavirus diarrhea among children younger than 5 years. JAMA Pediatr.

[2] Grimwood K, Lambert SB, Milne RJ (2010). Rotavirus infections and vaccines: burden of illness and potential impact of vaccination. Paediatr Drugs.

[3] Heymann DL (2015). Control of communicable disease manual.

[4] Crawford SE, Ramani S, Tate JE, Parashar UD, Svensson L, Hagbom M, Franco MA, Greenburg HB, O’Ryan M, Kang G, Desselberger U, Estes MK (2017). Rotavirus infection. Nat Rev Dis Primers.

[5] Esona MD, Gautam R (2015). Rotavirus. Clin Lab Med.

[6] World Health Organization (2013). Rotavirus vaccines; WHO position paper – January 2013. Wkly Epidemiol Rec.

[7] Ruiz-Palacios GM, Perez-Schael I, Velazquez FR, Abate H, Breuer T, Clemens SC, Cheuvart B, Espinoza F, Gilard P, Innis BL, Cervantes Y, Linhares AC, Lopez P, Macias-Parra M, Ortega-Barria E, Richardson V, Rivera-Medina DM, Rivera L, Salinas B, Pavia-Ruz N, Salmeron J, Ruttimann R, Tinoco JC, Rubio P, Nunez E, Guerrero ML, Yarzabal JP, Damaso S, Tornieporth N, Saez-Llorens X, Vergara RF, Vesikari T, Bouchenooghe A, Clemens R, De Vos B, O’Ryan M (2006). Human Rotavirus Vaccine Study Group. Safety and efficacy of an attenuated vaccine against severe rotavirus gastroenteritis. N Engl J Med.

[8] Vesikari T, Matson DO, Dennehy P, Van Damme P, Santosham M, Rodriguez Z, Dallas MJ, Heyse JF, Goveia MG, Black SB, Shinefield HR, Christie CD, Ylitalo S, Itsler RF, Coia ML, Onorato MT, Adeyi BA, Marshall GS, Gothefors L, Campens D, Karvonen A, Watt JP, O’Brien KL, DiNubile MJ, Clark HF, Boslego JW, Offit PA (2006). Heaton PM; rotavirus efficacy and safety trial (REST) study team. Safety and efficacy of a pentavalent human-bovine (WC3) reassortant rotavirus vaccine. N Engl J Med.

[9] Isanaka S, Guindo O, Langendorf C, Matar Seck A, Pilkaytis BD, Sayinzoga-Makombe N, McNeal MM, Meyer N, Adehossi E, Djibo A, Jochum B, Grais RF (2017). Efficacy of a low-cost, heat-stable oral rotavirus vaccine in Niger. N Engl J Med.

[10] Kulkarni PS, Desai S, Tewari T, Kawade A, Goyal N, Garg BS, Kumar D, Kanungo S, Kamat V, Kang G, Bavdekar A, Babji S, Juvekar S, Manna B, Dutta S, Angurana R, Dewan D, Dharmadhikari A, Zade JK, Dhere RM, Fix A, Power M, Uprety V, Parulekar V, Cho I, Chandola TR, Kedia VK, Raut A, Flores J (2017). SII BRV-PV author group. A randomized phase III clinical trial to assess the efficacy of a bovine-human reassortant pentavalent rotavirus vaccine in Indian infants. Vaccine.

[11] Bhandari N, Rongsen-Chandola T, Bavdekar A, John J, Antony K, Taneja S, Goyal N, Kawade A, Kang G, Rathore SS, Juvekar S, Muliyil J, Arya A, Shaikh H, Abraham V, Vrati S, Proschan M, Kohberger R, Thiry G, Glass R, Greenberg HB, Curlin G, Mohan K, Harshavardhan GV, Prasad S, Rao TS, Boslego J, Bhan MK, the India Rotavirus Vaccine Group (2014). Efficacy of a monovalent human-bovine (116E) rotavirus vaccine in Indian infants: a randomised, double-blind, placebo-controlled trial. Lancet.

[12] Burnett E, Jonestellar CL, Tate JE, Yen C, Parashar UD (2017). Global impact of rotavirus vaccination on childhood hospitalizations and mortality from diarrhea. J Infect Dis.

[13] Weldegebriel G, Mwenda JM, Chakauya J, Daniel F, Masresha B, Parashar UD, Tate JE (2018). Impact of rotavirus vaccine on rotavirus diarrhoea in countries of east and southern Africa. Vaccine.

[14] Bar-Zeev N, Jere KC, Bennett A, Pollock L, Tate JE, Nakagomi O, Iturriza-Gomara M, Costello A, Mwansambo C, Parashar UD, Heyderman RS, French N (2016). Cunliffe NA; vaccine effectiveness and disease surveillance Programme, Malawi (VACSURV) consortium. Population impact and effectiveness of monovalent rotavirus vaccination in urban Malawian children 3 years after vaccine introduction: ecological and case-control analyses. Clin Infect Dis.

[15] Chandran A, Fitzwater S, Zhen A, Santosham M (2010). Prevention of rotavirus gastroenteritis in infants and children: rotavirus vaccine safety, efficacy, and potential impact of vaccines. Biologics.

[16] Velasquez DE, Parashar U, Jiang B (2018). Decreased performance of live attenuated, oral rotavirus vaccines in low-income settings: causes and contributing factors. Expert Rev Vaccines.

[17] UNICEF (2005). Pacific office. Kiribati Islands. A situation analysis of children, women and youth.

[18] Liu L, Oza S, Hogan D, Perin J, Rudan I, Lawn JE, Cousens S, Mathers C, Black RE (2015). Global, regional, and national causes of child mortality in 2000-13, with projections to inform post-2015 priorities: an updated systematic analysis. Lancet.

[19] Tabunga T, Utiera M, Tekoaua R, Tibwe T, Tira T, Toatu T, Duituturaga SE, Nilles E, Craig A (2014). Response to a large rotavirus outbreak on South Tarawa, Kiribati, 2013. Western Pac Surveill Response J.

[20] World Health Organization. WHO vaccine-preventable diseases: monitoring system. 2018 global summary. WHO UNICEF estimates time series for Kiribati (KIR). http://apps.who.int/immunization_monitoring/globalsummary/estimates?c=KIR Accessed on 7th February 2019.

[21] Burnett E, Tate JE, Kirkwood CD, Nelson EAS, Santosham M, Steele AD, Parashar UD (2018). Estimated impact of rotavirus vaccine on hospitalizations and deaths from rotavirus diarrhea among children <5 in Asia. Expert Rev Vaccin.

[22] UN Inter-agency Group for Child Mortality Estimation. Levels & trends in child mortality, Report 2015. United Nations Children’s Fund, 2015. p. 23.

[23] Creek TL, Kim A, Lu L, Bowen A, Masunge J, Arvelo W, Smit M, Mach O, Legwaila K, Motswere C, Zaks L, Finkbeiner T, Povinelli L, Maruping M, Ngwaru G, Tebele G, Bopp C, Puhr N, Johnston SP, Dasilva AJ, Bern C, Beard RS, Davis MK (2010). Hospitalization and mortality among primarily nonbreastfed children during a large outbreak of diarrhea and malnutrition in Botswana, 2006. J Acquir Immune Defic Syndr.

[24] Gordon DM, Frenning S, Draper HR, Kokeb M (2013). Prevalence and burden of diseases presenting to a general pediatrics ward in Gondar. Ethiopia J Trop Pediatr.

[25] Lenters LM, Wazny K, Webb P, Ahmed T, Bhutta ZA (2013). Treatment of severe and moderate acute malnutrition in low- and middle-income settings: a systematic review, meta-analysis and Delphi process. BMC Public Health.

[26] World Health Organization. Children: reducing mortality. Geneva Switzerland, 2016. http://www.who.int/news-room/fact-sheets/detail/children-reducing-mortality Accessed on 19th November 2018.

